# Comparative study of three commonly used continuous deterministic methods for modeling gene regulation networks

**DOI:** 10.1186/1471-2105-11-459

**Published:** 2010-09-14

**Authors:** Martin T Swain, Johannes J Mandel, Werner Dubitzky

**Affiliations:** 1 Roche Diagnostics GmbH, Nonnenwald 2, 82372 Penzberg, Germany; 2 University of Ulster, School of Biomedical Sciences, Cromore Road, Coleraine BT52 1SA, Co. Londonderry, UK

## Abstract

**Background:**

A gene-regulatory network (GRN) refers to DNA segments that interact through their RNA and protein products and thereby govern the rates at which genes are transcribed. Creating accurate dynamic models of GRNs is gaining importance in biomedical research and development. To improve our understanding of continuous deterministic modeling methods employed to construct dynamic GRN models, we have carried out a comprehensive comparative study of three commonly used systems of ordinary differential equations: The *S-system (SS), artificial neural networks (ANNs), and the general rate law of transcription (GRLOT) *method. These were thoroughly evaluated in terms of their ability to replicate the reference models' regulatory structure and dynamic gene expression behavior under varying conditions.

**Results:**

While the ANN and GRLOT methods appeared to produce robust models even when the model parameters deviated considerably from those of the reference models, SS-based models exhibited a notable loss of performance even when the parameters of the reverse-engineered models corresponded closely to those of the reference models: this is due to the high number of power terms in the SS-method, and the manner in which they are combined. In cross-method reverse-engineering experiments the different characteristics, biases and idiosynchracies of the methods were revealed. Based on limited training data, with only one experimental condition, all methods produced dynamic models that were able to reproduce the training data accurately. However, an accurate reproduction of regulatory network features was only possible with training data originating from multiple experiments under varying conditions.

**Conclusions:**

The studied GRN modeling methods produced dynamic GRN models exhibiting marked differences in their ability to replicate the reference models' structure and behavior. Our results suggest that care should be taking when a method is chosen for a particular application. In particular, reliance on only a single method might unduly bias the results.

## 1 Background

Regulation of gene expression (or gene regulation) refers to processes that cells use to create functional gene products (RNA, proteins) from the information stored in genes (DNA). These processes range from DNA-RNA transcription to the post-translational modification of proteins. Gene regulation is essential for life as it increases the versatility and adaptability of an organism by allowing it to express protein when needed. While aspects of gene regulation are well understood, many open research questions still remain [1]. Due to the wide availability of well-characterized components from biological gene networks, the stage has been set for mathematical modeling and computational simulation of *gene regulatory networks *(GRNs). The modeling of biomedical phenomena is inspired by the approach taken in physics. In physics, models (theories) are frequently constructed to explain existing data, then predictions are made, which again are compared to new data. If a sufficient correspondence exists, it is claimed that the phenomenon has been understood. A model should not be a *black box*, but should be interpretable in some way. Ideally, the model components and elements should have an interpretation in the real world consistent with the existing knowledge. At the same time a model involves a simplification of the real world. The ability to construct, analyze, and interpret qualitative and quantitative *GRN models *is becoming increasingly important tool in studying gene regulation [2]. Because of its potential to help improve our understanding of gene regulation, modeling and simulation of GRNs has received considerable interest in the bioinformatics and computational biology communities. Many mathematical and computational techniques have been proposed in recent years. In practice, different mathematical techniques generate models with different properties and features. Therefore, it is very important for users to understand the merits and limitations of these approaches.

The dynamic behavior and regulatory interactions of genes can be revealed by time-series experiments, that is, experiments that measure the expression of multiple genes over time [3-6]. As this type of experimental data becomes more readily available, mathematical modeling and computational simulation become an important tool for investigating the structure and time-dependent behavior of GRNs. In contrast to static gene expression data, the modeling and simulation approach allows the determination of stable states in response to a condition or stimulus as well as the identification of pathways and networks that are activated in the process [7]. Besides logical [8-10] and stochastic [11] modeling approaches, various continuous modeling methods capable of capturing such complex behavior deterministically are commonly used. A range of mathematical methods facilitating the reverse-engineering of quantitative, dynamic GRN models from time-series expression data have been reported in the literature [12].

Our study focused primarily on *continuous deterministic *simulation methods used to model and simulate GRNs on the basis of time-variant gene expression data. The approaches considered in our study concentrate on GRN abstractions that ignore intricate intermediate biological processes of cellular gene regulation, such as splicing, capping, translation, binding and unbinding [13]. Because of their importance in the field and their common use, we have compared the following three mathematical methods:

• The *S-system *(SS) method [14];

• The *artificial neural network *(ANN) method [15];

• The *general rate law of transcription *(GRLOT) method [16].

All three methods describe dynamic systems by non-linear equations, yet they are different in the way they process regulatory signals and, hence, in how they model gene expression and gene regulation. In addition, these methods employ different model parameters to represent gene-regulatory mechanisms. Thus, each method conveys a different conceptual view of the underlying GRN. Our comparison of the three methods was guided by the following questions:

1. How do the methods fare in terms of their ability to estimate model parameters from dynamic gene expression data?

2. How do the methods fare in terms of their ability to accurately predict dynamic gene expression data?

3. To what extent are the methods interchangeable?

4. Are there any inherent biases or limitations in the individual methods?

To answer the above questions we developed artificial data sets based on a number of model networks that were selected because they either incorporate motifs frequently seen in GRNs, or because they have been specifically discussed in the literature in the context of the three mathematical methods of interest. The advantages of using artificial data sets is due to the ease, speed and flexibility with which different experiments can be performed and evaluated, allowing validation of the methods under a range of tightly controlled conditions which would be very difficult, expensive and time-consuming to perform with real biological data. However, computer generated data sets feature important differences when compared to real biological data [17,18], and so we have also included a recent biological data set generated by Cantone *et al. *specifically for *in vivo *assessment of reverse-engineering and gene network modelling approaches [19]. The reverse-engineering experiments we have performed on this biological network play an important role in demonstrating that the results we have obtained from our model networks are applicable to real biological networks.

**Figure 1:**
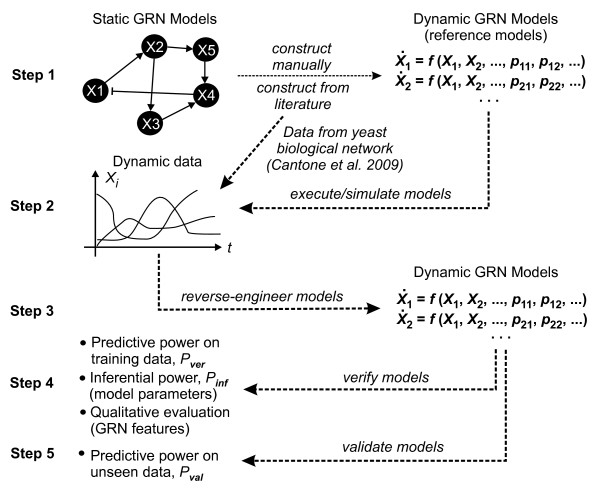
**Study design**. Summary of experimental set-up, involving manual construction of dynamic GRN models (Step 1), simulation/execution of these models to generate various dynamic gene expression data sets (Step 2), the automated reverse-engineering of dynamic GRN models from these data (Step 3), and in silico experimentation to verify (Step 4) and validate (Step 5) the reverse-engineered models.

Our study is relatively comprehensive and involved. To facilitate a clear understanding of our study design, we outline the main logical steps below. The diagram in Figure 1 summarizes the workflow of our experimental set-up. More detail of the data, models and results corresponding to the steps in the study is provided in the main body of the paper, in Section 2 and Section 4.

1. To investigate a range of different conditions, we obtain two time-series data sets for a biological network from the literature [19], and we define and construct five artificial network models and set their parameters and initial expression levels.

2. We wish to explore how the quantity of available data influences the models produced by reverse-engineering. Therefore, for each artificial network model we generated two sets of data -*sparse and detailed data *- using each mathematical method. Sparse data is based on a single model simulation, detailed data is based on five simulations each with a different set of initial condition.

3. Here we perform the reverse-engineering of dynamic GRN models from the artificial and biological data sets using an evolutionary algorithm [20]. In order to highlight differences and idiosynchracies between and within the models, we apply each mathematical method to all the available data sets. A large number of models are generated in this step, investigating combinations of different data sets, experimental scenarios, and mathematical methods.

4. This step is designed to assess specific aspects of the models, and to elucidate the extent to which the reverse-engineered models are able to discover the original GRN models and their parameters, or to reproduce their dynamic behavior according to the relevant biological or artificial time-series data sets.

5. The final step is designed to investigate if the GRN models are able to predict how the network will behave under new conditions, and if the mathematical methods are all able to correctly capture the behavior of the GRNs: if this is true then all our reverse-engineered models should behave in a similar fashion under new conditions and make similar predictions about the network behavior.

This paper is structured as follows: first we outline the mathematical methods, then we discuss their different characteristics, strengths and weaknesses, followed by the different GRN models to which we apply them. We then outline our results and relate them to the experimental workflow. Before concluding we give a detailed discussion of differences between the mathematical methods as shown by our results.

### 1.1 GRN modeling methods

The non-linear differential equations of the three modeling methods investigated in this study describe the mutual activating and repressing influences of genes in a GRN at a high-level of abstraction. In particular, it is assumed that the rate of gene expression depends exclusively on the concentration of gene products arising from the nodes (genes) of the GRN. This means that the influence of other molecules (e.g., transcription factors) and cellular processes (translation) is not taken into account directly. Even with these limitations, dynamic GRN models of this kind can be useful in deciphering basic aspects of gene-regulatory interactions. The three methods we have studied have been widely used to model dynamic GRNs. One major advantage of all three methods lies in their simple homogeneous structures, as this allows the settings of parameter discovering software to be easily customized for these structures. In addition, all three modeling methods either already have the potential to describe additional levels of detail, or their structures can be easily extended for this purpose.

The three methods describe dynamic GRN models by means of a system (or set) of ordinary *differential equations*. For a GRN comprising *N *genes, *N *differential equations are used to describe the dynamics of *N *gene product concentrations, *X_i _*with *i *= 1, ..., *N*. In all three methods, the expression rate *dX_i_/dt *of a gene product concentration may depend on the expression level of one or more gene products of the genes *X_j_*, with *j *= 1, ..., *N*. Thus, the gene product concentration *X_i _*may be governed by a self-regulatory mechanism (when *i *= *j*), or it may be regulated by products of other genes in the GRN. The three modeling methods differ in the way they represent and calculate expression rates. Before discussing such differences, we introduce the three modeling methods in some detail.

#### 1.1.1 The artificial neural network (ANN) method

Vohradsky [15] introduced ANNs as a modeling method capable of describing the dynamic behavior of GRNs. The way this method represents and calculates expression rates depends on the weighted sum of multiple regulatory inputs. This additive input processing is capable of representing logical disjunctions. The expression rate is restricted to a certain interval where a sigmoidal transformation maps the regulatory input to the expression interval. ANNs provide an additional external input which has an influence on this transformation in that it can regulate the sensitivity to the summed regulatory input. Finally, the ANN method defines the degradation of a gene product on the basis of standard mass-action kinetics.

Formally, the ANN method is defined as:

(1) $$\begin{array}{cc} \frac{dX_{i}}{dt}=v_{i}\cdot f(\sum_{j=1}^{N}w_{ij}X_{j}-\vartheta_{i})-k_{i}X_{i} & \vartheta_{i}v_{i}k_{i}>0 \end{array}$$

The parameters of the ANN method have the following biological interpretations:

*N*: Number of genes in the GRN to be modeled. The genes of the GRN are indexed by *i *and *j*, where *i*, *j *= 1, ..., *N*.

*v_i_*: Maximal expression rate of gene *i*.

*w_ij_*: The connection weight or strength of control of gene *j *on gene *i*. Positive values of *w_ij _*indicate activating influences while negative values define repressing influences.

ϑ_*i*_: Influence of external input on gene *i*, which modulates the gene's sensitivity of response to activating or repressing influences.

*f*: Represents a non-linear sigmoid transfer function modifying the influence of gene expression products *X_j _*and external input *ϑ_i _*to keep the activation from growing without bounds.

*k_i_*: Degradation of the *i*-th gene expression product.

The mathematical properties of the ANN method have been well studied because it is a special case of a recurrent neural network [15]. In particular, the symmetry of the matrix of connection weights *w_ij _*influences whether the network dynamics are oscillatory or whether they converge on a steady (or even chaotic) state. High positive or negative values of the external input, *ϑ_i_*, reduce the effect of the connection weights. This is explored in Case D where *ϑ_i _*has been interpreted as a delay to the reaction kinetics of the transcriptional machinery [15].

#### 1.1.2 The S-system (SS) method

Savageau [14] proposed the *synergistic system *or *S-system *(*SS*) as a method to model molecular networks. When modeling GRNs with the SS method, the expression rates are described by the difference of two products of power-law functions, where the first represents the activation term and the second the degradation term of a gene product *X_i_*. This *multiplicative *input processing can be used to define logical conjunctions for both the regulation of gene expression processes and for the regulation of degradation processes. The SS method has no restrictions in the gene expression rates and thus does not implicitly describe saturation.

Formally, the SS method is defined as:

(2) $$\frac{dX_{i}}{dt}=\alpha_{i}\prod_{j=1}^{N}X_{j}^{g_{ij}}-\beta_{i}\prod_{j=1}^{N}X_{j}^{h_{\;ij}}\;\alpha_{i},\;\beta_{i}>0,\;g_{ij},\;h_{ij}\in ℜ.$$

The parameters of the SS method have the following biological interpretations:

*N*: Number of genes in the GRN to be modeled. The genes of the GRN are indexed by *i *and *j*, where *i*, *j *= 1, ..., *N*.

*α_i_*: Rate constant of activation term; in SS GRN models, all activation (up-regulation) processes of a gene *i *are aggregated into a single activation term.

*β_i_*: Rate constant of degradation term; in SS GRN models, all degradation processes of a gene *i *are aggregated into a single degradation term.

*g_ij _*, *h_ij_*: Exponential parameters called *kinetic order*. These parameters describe the interactive influences of gene *j *on gene *i*. Positive values of *g_ij _*indicate an activating influence on the expression of gene *i*, whereas inhibiting influences are represented by negative values. Similarly, positive values of *h_ij _*indicate increasing degradation of the gene product *X_i_*, whereas decreasing degradation is represented by negative values.

The parameters used in SS models have a clear physical meaning and can be measured experimentally [21], yet they describe phenomenological influences, as opposed to stoichiometric rate constants in *general mass action *(*GMA*) systems [22]. The SS method generalizes mass-action kinetics by aggregating all individual processes into a single activation and a single degradation term (per gene). In contrast, the GMA system defines all individual processes *k *with *k *= 1, ..., *R *with the sum of power-law functions [23] according to:

(3) $$\frac{dX_{i}}{dt}=\sum_{k=1}^{R}\alpha_{ik}\prod_{j=1}^{N}X_{j}^{g_{ijk}}-\sum_{k=1}^{R}\beta_{ik}\prod_{j=1}^{N}X_{j}^{h_{ijk}}\;\alpha_{ik},\beta_{ik}>0,\;\;g_{ijk},h_{ijk}\in ℜ.$$

The parameters of the GMA system have the following biological interpretations:

*α_i_*: Rate constant of activation process *k*.

*β_ik_*: Rate constant of degradation process *k*.

*g_ijk_*: Exponential parameter called *kinetic order *describing the interactive influence of *X_j _*on gene *i *of process *k*.

*h_ijk_: *Exponential parameter called kinetic order describing the interactive influence of *X_j _*on gene *i *of process *k*.

#### 1.1.3 The general rate law of transcription (GRLOT) method

The GRLOT method has been used to generate benchmark time-series data sets to facilitate the evaluation of different reverse-engineering approaches [16,24]. GRLOT models multiply individual regulatory inputs. Activation and inhibition are represented by different functional expressions that are similar to Hill kinetics, which allow the inclusion of cooperative binding events [16]. Identical to the ANN, the degradation of gene products is defined via mass-action kinetics.

Formally, the GRLOT method is defined as:

(4) $$\frac{dX_{i}}{dt}=v_{i}\prod_{j}(\frac{Ki_{j}^{n_{j}}}{I_{j}^{n_{j}}+Ki_{j}^{n_{j}}})\times \prod_{k}(\frac{A_{k}^{n_{k}}}{A_{k}^{n_{k}}+Ka_{k}^{n_{k}}})-k_{i}X_{i}\;v_{i},Ki_{j},Ka_{j},k_{i}>0,$$

The parameters of the GRLOT method have the following biological interpretations:

*v_i_*: Maximal expression rate of gene *i*.

*I_j_*: Inhibitor (repressor) *j*.

*A_k_*: Activator *k*; the number of inhibitors *I*, and the number of activators *A *can be related to the total number of genes by *I *+ *A ≤ N*.

*Ki_j_*: Concentration at which the influence of inhibitor *j *is half of its saturation value.

*Ka_k_*: Concentration at which the influence of activator *k *is half of its saturation value.

*n_j _*, *n_k_*: Regulate the sigmoidicity of the interaction behavior in the same way as Hill coefficients in enzyme kinetics.

*k_i_*: Degradation of the *i*-th gene expression product.

### 1.2 Systematic biases in the ANN, SS and GRLOT methods

Major differences among the three methods for GRN modeling can be found in the limitation of expression rates, regulation of degradation processes, in the processing of multiple inputs, and in the interpretation of model parameters.

#### Limitation of expression rates

The SS method has no restrictions on expression rates, *dX_i_/dt*, while the ANN and GRLOT methods restrict expression rates to the interval [0, *v_i_*] through a sigmoid function. The three methods have in common that concentration changes are not synchronized, that is, the formation or synthesis of a molecule does not necessarily entail the reduction or degradation of other molecules. (In particular, this can quickly lead to unrealistic dynamics in SS models as singularities may arise when modeling inhibition and the inhibitor reaches a near-zero concentration.) However, the expression concentration of a gene product must reach a saturation level at some point and a sigmoid expression rate response is thought to act as the molecular switch that models this behavior [25].

#### Regulation of degradation processes

The SS method covers a wider spectrum of regulatory targets since SS models are able to describe the regulation of both transcription and degradation processes.

#### Multiple input processing

GRN models based on the ANN method weight and sum multiple regulatory inputs. This contrasts with dynamic GRN models based on the SS and GRLOT methods, where regulatory inputs are exponentiated and multiplied. The input processing in SS and GRLOT models certainly accord better with the fundamentals of reaction kinetics and collision theory (chemical reaction rates correlate with the multiplied concentrations of the reactants) than ANN models. Nevertheless, none of the three methods follow strictly the chemical reaction processes as a single kinetic equation typically comprises several processes. Instead, all methods are designed to be flexible enough to find phenomenological expressions for their approximations of experimentally observed behavior [15,22]. In particular, for the SS method, we have found that the unrestricted expression rates coupled with multiple input processing based on multiplied exponentials is a serious flaw, frequently leading to extreme sensitivity and unstable dynamics (as we describe in Section 2).

#### Parameter interpretation

The different parameters used in the three methods describe activating (inducing) and inhibiting (repressing) interactions among the genes of a GRN:

• In the ANN method, the parameters *w_ij _*can be intuitively used to define activation and inhibition by assigning positive or negative values, respectively. Here, small absolute values indicate a minor impact on the transcription process (or a missing regulatory interaction) and large absolute values indicate a correspondingly major impact on the transcription process. However, one should be aware that the multiple regulatory inputs required in the case of co-regulation can compensate each other due to their additive input processing.

• The GRLOT method uses two inverse functional forms to describe activating and inhibiting influences both of which involve two parameters. For example, in the case of activation, each individual dependency is described with a Hill equation where the Hill exponent *n_ij _*determines the sigmoid response curve to varying concentration levels of the regulating molecule. Furthermore, the second parameter, *Ka_k_*, allows the concentration of the regulator molecule to be specified, which gives half of its maximal influence on the expression rate. Consequently, high exponent values together with low *Ka_k _*values, defined for a regulator molecule, indicate that already low concentration levels have a strong activating influence on the transcription process while, on the other hand, low exponent values in combination with high *Ka_k _*values require high concentration levels of the regulator molecule for effective activation.

• The behavior of SS models is mainly determined by the exponent values *g_ij _*and *h_ij _*. Similar to the ANN method, high absolute values define strong influence, whereas small absolute values indicate weak influence. However, the dynamics can become particularly complicated when describing inhibiting dependencies using negative exponent values. Here, the effect depends strongly on the actual concentration ranges of the inhibitor which can introduce singular behavior at near-zero concentrations.

These differences make each method unique in its application to modeling GRNs. Table 1 provides a summary of the characteristics of the three modeling methods.

**Table 1:** Method characteristics

Characteristic	ANN	SS	GRLOT
number of parameters	N * (*N *+ 3)	N * (2 * *N *+ 2)	N * (2 * *N *+ 1)

limitation (saturation and sigmoidicity) of expression rates	yes	no	yes

Processing of multiple signals	additive, logical disjunction (OR)	multiplicative, logical conjunction (AND) corresponds well with collision theory	multiplicative, logical conjunction (AND) corresponds well with collision theory

regulation of degradation processes	no	yes	no

singularities	no	yes	no

Characteristics of the artificial neural network (ANN), the S-system (SS) and the General Rate Law Of Transcription (GRLOT) methods.

### 1.3 Manually constructing case study models

Due to the limited number of time points at which gene expression measurements are typically made, the reverse-engineering of GRNs usually constitutes an *under-determined *problem. Essentially, this means that there are more parameters to estimate than there are measurements [26,27]. Although hundreds of gene activities can be measured simultaneously with microarray experiments, the number of (time-dependent) data points established for each gene is typically small, leading to highly under-determined systems. As a consequence, multiple solutions can be found through reverse-engineering which are able to fit the available data very well, yet which are very weak in their ability to predict dynamic activity under different conditions to those initially explored [28].

Therefore, in order to reduce the number of parameters when performing a modeling study, a first step may involve analyzing gene expression profiles and performing clustering of genes exhibiting similar dynamics [29]. However, the relationships between co-regulated genes are very complex [30] and it is important to identify the key genes that act as regulators to the clusters of co-expressed genes [31]. Once the gene regulators are identified they may be used to construct simplified network models that are useful for reverse-engineering [32]. For instance, Kimura and colleagues [33] built a quantitative model comprising 24 gene groups found in 612 putative open reading frames measured with UvrA gene disruptant experiments in Thermus thermophilus HB8 strains, while Guthke and co-workers [34] clustered a total of 1336 genes into six clusters for dynamic network reconstruction from gene expression data. The latter example was used to investigate the immune response during bacterial infection of peripheral blood mononuclear cells.

**Figure 2:**
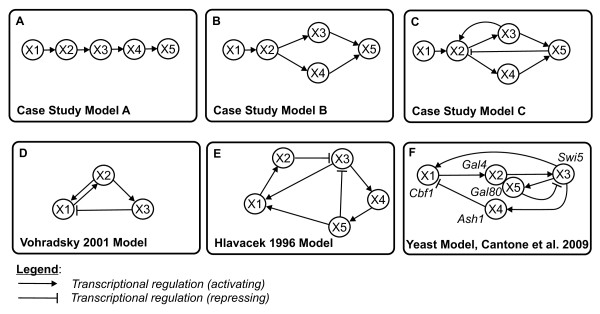
**Case study models**. The six GRN models A, B, C, D, E and F used in this comparative study.

With all three mathematical systems introduced in Section 1.1, we construct a set of case study models comprising only a small number of genes similar to the gene clusters used in many modeling studies (Figure 2). Furthermore, they describe network motifs comprising regulatory cascades, single input modules, multiple input modules and multi-component loops [35,36]. By first manually constructing and simulating these GRNs, and then performing reverse-engineering on the simulated data, we can greatly limit the problems associated with the poor information content of under-sampled data. The case study models are shown in Figure 2.

#### 1.3.1 Case studies A, B and C

Each of these three cases is modeled using the ANN, SS and GRLOT method. The parameters of each model are manually chosen, though we ensured that the models produce plausible dynamics.

In all models describing the cases A, B and C, we circumscribed regulatory operations to expression processes since the regulation of degradation processes can be only described with the SS method. For each gene product *X_i _*we therefore set in all constructed models the degradation rates *k_i _*(*β_i _*in the SS method) to 0.3. In all SS models we set the exponent values *h_ij _*to 1. This allows us to concentrate on the transcriptional part of the different methods.

• Case A: This static GRN model describes a four-step gene activation cascade starting from gene X1 up to gene X5. To generate harmonic dynamics with models of case A, we defined in all three methods (ANN, SS and GRLOT) constant signal propagation in the cascades by using identical parameter values within the parameter sets.

• Case B: With this model, we describe again a gene activation cascade, however, this case incorporates signal branching (multiple output) and co-regulatory relationships (multiple input) into the GRN. Here, gene X2 activates gene X3 and X4, both of which stimulate (activate) the expression of gene X5. We defined asymmetric signal branching such that the regulatory impact between X2 and X3 is different from the regulatory impact between X2 and X4. Furthermore, the co-regulated gene X5 is more sensitive to signals originating from X4 than to those of X3.

• Case C: The gene network described in the static GRN model C extends the GRN described in scenario B by incorporating multi-component loops, a positive feedback loop between gene X2 and gene X3, and a negative feedback loop between the genes X2 and X5. The models describing case C simply extend the parameter sets of case B with parameters defining stimulation between X3 and X2 and inhibition between X2 and X5. Thus, all parameter sets of case C contain two closed-loop relationships, one positive feedback loop between X2 and X3, and one negative feedback loop between X2 and X5.

The common characteristics shared by the different regulatory parameter matrices (arising from *N *coupled differential equations that represent a dynamic GRN model) are summarized as follows:

1. Case A: Uniform degradation rate (0.3), and constant signal propagation.

2. Case B: Uniform degradation rate (0.3), asymmetric signal branching and asymmetric co-regulation.

3. Case C: Uniform degradation rate (0.3), negative and positive feedback loops.

We use these characteristics as evaluation criteria in our qualitative analysis of the accuracy of each reverse-engineered model (Section 4.5.3). All model parameters are provided in Table 2. The increasing complexity of the scenarios with their different regulatory mechanisms allowed us to gradually analyze and compare the individual modeling systems.

**Table 2:** Method parameters for case studies

	The SS method							The ANN method							
	***α***	***β***	**X1**	**X2**	**X3**	**X4**	**X5**	***v***_***i***_	***θ***	***k***	**X1**	**X2**	**X3**	**X4**	**X5**

Case A															

X1	0.0	0.3	-	-	-	-	-	1.0	4.0	0.3	-	-	-	-	-

X2	1.0	0.3	2.0	-	-	-	-	1.0	4.0	0.3	5.0	-	-	-	-

X3	1.0	0.3	-	2.0	-	-	-	1.0	4.0	0.3	-	5.0	-	-	-

X4	1.0	0.3	-	-	2.0	-	-	1.0	4.0	0.3	-	-	5.0	-	-

X5	1.0	0.3	-	-	-	2.0	-	1.0	4.0	0.3	-	-	-	5.0	-

Case B															

X1	0.0	0.3	-	-	-	-	-	1.0	4.5	0.3	-	-	-	-	-

X2	1.0	0.3	1.5	-	-	-	-	1.0	4.5	0.3	7.0	-	-	-	-

X3	1.0	0.3	-	2	-	-	-	1.0	4.5	0.3	-	6.0	-	-	-

X4	1.0	0.3	-	3	-	-	-	1.0	4.5	0.3	-	4.0	-	-	-

X5	1.0	0.3	-	-	2	3	-	1.0	4.5	0.3	-	-	4.0	6.0	-

Case C															

X1	0.0	0.3	-	-	-	-	-	1.0	4.5	0.3	-	-	-	-	-

X2	1.0	0.3	1.5	-	0.6	-	-0.3	1.0	4.5	0.3	7.0	-	7.0	-	-8.0

X3	1.0	0.3	-	2	-	-	-	1.0	4.5	0.3	-	6.0	-	-	-

X4	1.0	0.3	-	3	-	-	-	1.0	4.5	0.3	-	4.0	-	-	-

X5	1.0	0.3	-	-	2	3	-	1.0	4.5	0.3	-	-	4.0	6.0	-

The GRLOT method															

	V	k	X1 n	X1 ki	X2 n	X2 ki	X3 n	X3 ki	X4 n	X4 ki	X5 n	X5 ki			

Case A															

X1	0.0	0.3	-	-	-	-	-	-	-	-	-	-			

X2	1.0	0.3	1.5	1.1	-	-	-	-	-	-	-	-			

X3	1.0	0.3	-	-	1.5	1.1	-	-	-	-	-	-			

X4	1.0	0.3	-	-	-	-	1.5	1.1	-	-	-	-			

X5	1.0	0.3	-	-	-	-	-	-	1.5	1.1					

Case B															

X1	0.0	0.3	-	-	-	-	-	-	-	-	-	-			

X2	1.0	0.3	1.5	0.8	-	-	-	-	-	-	-	-			

X3	1.0	0.3	-	-	2.5	0.5			-						

X4	1.0	0.3	-	-	1.5	0.8		-							

X5	1.0	0.3	-	-	-	-	1.5	0.8	2.5	0.5					

Case C															

X1	0.0	0.3	-	-	-	-	-	-	-	-	-	-			

X2	1.0	0.3	1.5	0.8	-	-	3.5	0.1	-	-	-1.5	2.0			

X3	1.0	0.3	-	-	2.5	0.5	-	-	-	-	-	-			

X4	1.0	0.3	-	-	1.5	0.8	-	-	-	-	-	-			

X5	1.0	0.3	-	-	-	-	1.5	0.8	2.5	0.5	-	-			

Parameters for each of the SS, ANN and GRLOT methods as used in case study models A, B and C.

#### 1.3.2 Case studies D and E

**Figure 3:**
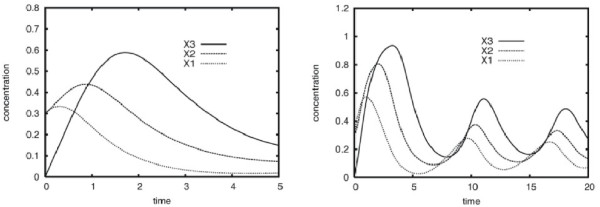
**Dynamics of a 3-gene model**. Various dynamics of the 3-gene model taken from Vohradsky (2001) [15], with *ϑ*_1 _= 3.0 (left) and *ϑ*_1 _= 1.0 (right). Small variations in the ANN parameter *ϑ_i _*cause different dynamics with constant topology parameters.

The following two GRN scenarios (D and E) were taken from the literature [15,21]. These are depicted in Figure 2:

• Case D (V): The 3-gene static GRN model describing case D was used elsewhere to discuss the influence of the external input parameter *ϑ_i _*on the dynamics of an ANN GRN model [15]. In this example *ϑ_i _*is interpreted as delaying the reaction kinetics. Figure 3 shows two dynamic gene expression behaviors obtained by small variations in this parameter. This 3-gene GRN model comprises a positive and negative feedback regulation where products of gene X1 activate the expression of gene X2 and products of gene X2 stimulate the expression of gene X3 and X1. Gene X3 inhibits the expression of gene X1.

• Case E (HS): The fifth GRN system is the 5-gene static GRN model used by Hlavacek and Savageau [21]. This model is frequently used to evaluate reverse-engineering algorithms in combination with the SS method [27,33,37]. The dynamic behavior of this model is strongly influenced by singularities allowed by the SS method. In this scenario, singularities can be caused by the inhibitory dependency between gene X5 and X3 when the concentration level of X5 reaches zero.

We use the two cases (D and E), which were specifically generated for the ANN and SS methods, respectively, to provide additional evidence for the idiosyncratic behavior of the investigated modeling methods.

#### 1.3.3 Case study F

This biological GRN was included in this study to ensure that the results we obtain from our artificial networks can be transferred to real biological systems. It was constructed synthetically in yeast by Cantone *et al. *[19] to facilitate an in vivo assessment of various reverse-engineering and gene network modelling approaches, including approaches based on ordinary differential equations.

Cantone *et al. *present expression profiles of the network genes after a shift from glucose- to galactose-raffinose-containing medium: this is called the *switch-on *time series; and after a shift from galactose-raffinose to glucose-containing medium: the *switch-off *time series. In this study we used the first 100 minutes of these two data sets, excluding the first 10 minute interval during which the washing steps and subsequent medium shift are performed. After 100 minutes the biological system is perturbed, and Cantone *et al *use time-delay terms to model this perturbation. We have not explored time-delay terms in our mathematical systems and so we do not model this perturbation.

The network includes a variety of regulatory interactions, thus capturing the behavior of larger eukaryotic gene networks on a smaller scale (Figure 2). It was designed to be minimally affected by endogenous genes and to transcribe its genes in response to galactose. While the yeast GRN appears relatively simple, it is actually quite articulated in its interconnections, which include regulator chains, single-input motifs, and multiple feedback loops, which are generated by the combination of transcriptional activators and repressors.

## 2 Results and Discussion

As we performed many different experiments, we first clarify how the different stages of our study contribute to our final set of results before discussing them in detail.

### 2.1 Results

Figure 1 describes the study design we adopted to compare the three modeling methods. Here we briefly describe the inputs and outputs of each step along with the relevant result tables and figures.

#### Step 1: Construction of reference models

*Input*: Three 5-gene static GRN models (models A, B, C), comprising regulatory cascades, single input modules, multiple input modules and multi-component loops [35,36]. Three models taken from the literature: a 3-gene static GRN model (D) [15], a 5-gene static GRN model (E) [21], and a 5-gene biological GRN (model F) synthetically generated in yeast [19].

*Output*: 13 dynamic GRN models:

• Nine 5-gene dynamic GRN models based on the static models A, B and C. For each static model, three dynamic models were constructed using the modeling methods ANN, SS and GRLOT, respectively.

• One 3-gene dynamic GRN model based on static model D, using the ANN method. The model parameters were taken directly from the literature [15].

• One 5-gene dynamic GRN model based on static model E, using the SS method. The model parameters were taken directly from the literature [21].

• Two 5-gene dynamic GRN models based on the switch-on and switch-off time series data sets presented in the literature [19].

#### Step 2: Generation of the artificial GRN expression data

*Input*: The 11 artificial GRN models from Step 1 (based on models A-E).

*Output*: 20 dynamic gene expression data sets:

• 11 *sparse *data sets based on simulating each GRN model with a single set of initial conditions.

• 9 *detailed *data sets from the first nine models of Step 1 (corresponding to the static GRN models A, B and C, respectively). Each data set consists of 5 time series each created with different initial conditions.

#### Step 3: Reverse-engineering of dynamic GRN models from generated data

*Input*: The output from Step 2, 20 dynamic gene expression data sets, plus the output from Step 1 for model F only, the switch-on and switch-off data sets: giving a total of 22 data sets.

*Output*: 66 dynamic GRN models. Each of the 22 data sets is reverse-engineered using the ANN, SS and GRLOT method, respectively.

#### Step 4: Verification of reverse-engineered GRN models

*Input*: 66 dynamic GRN models from Step 3.

*Output*: Calculation of training predictive power, *P_ver_*, the inferential power, *P_inf _*, and qualitative and visual comparison of GRN features:

• Execution of 60 dynamic GRN for models A-E from Step 3 to determine 60 *P_ver _*values, each reflecting how well the model is able to predict (fit) the data from which it was generated.

• Calculation of the inferential power, *P_inf _*, for 20 of the 60 models by comparing the reverse-engineered model parameters to the model parameters of the reference models from Step 1. This is possible only for model pairs, from Step 1 and Step 3, that share the same modeling method.

• Qualitative analysis of GRN features.

• Visual analysis of the models generated from the switch-on and switch-off data sets for the biological network (model F) as described in Section 2.2.1.

Table 3 and Table 4 summarize the results in terms of training (or verification) predictive power, *P_ver_*, and inferential power, *P_inf _*, determined for the reverse-engineered ANN, SS and GRLOT models, when reproducing the network dynamics. The results of the qualitative analysis are presented in Table 5.

**Table 3:** Reverse-engineering verification and validation results for A, B and C

	ANN data			SS data			GRLOT data		
	**Sparse data set**								

*P*_*inf*_	0.9620	0.9701	0.8562	0.9308	0.9994	0.9947	0.9546	0.4820	0.6046

*P*_*ver*_	0.9996	0.9797	0.9982	0.9998	0.9988	0.9973	0.9985	0.9941	0.9999

*P*_*val*_	0.9972	0.9870	0.9976	0.9953	0.8635	0.7878	0.9982	0.9927	0.9999

Δ *P*_*fit*_	**-0.0024**	**0.0073**	**-0.0016**	**-0.0045**	**-0.1353**	**-0.2095**	**-0.0003**	**-0.0014**	**0.0000**

	**Detailed data set**								

*P*_*inf*_	0.9896	0.9992	1.0000	1.0000	0.9999	1.0000	0.6704	0.7070	0.6432

*P*_*ver*_	0.9998	0.9999	1.0000	1.0000	1.0000	1.0000	0.9983	0.9978	0.9987

*P*_*val*_	0.9997	0.9999	1.0000	0.9978	0.9882	0.9891	0.9978	0.9972	0.9996

Δ*P*_*fit*_	**-0.0001**	**0.0000**	**0.0000**	**-0.0022**	**0.0118**	**0.0109**	**-0.0005**	**-0.0006**	**0.0009**

Summary of verification (Step 4) and validation (Step 5) results from reverse-engineering dynamic GRN models from data generated with the reference models A, B and C (Step 1). Here, only *same-method *results are shown - i.e., ANN model reverse-engineered with ANN method from data generated with ANN reference model, and so on. Results are given for cases A, B, and C for each method (columns below method name from left to right). For the *sparse *and *detailed *data sets, *P_inf _*and *P_ver _*first given for the reverse-engineered models, then *P_val _*for the predictions (averaged over 2 values for input increase and decrease). The Δ*P_fit _*(for data fitting) is the difference between *P_ver _*and *P_val _*scores.

**Table 4:** Reverse-engineering results with data fitting scores only

Sparse data		ANN data	SS data	GRLOT data
to ANN model	*P_ver_*	0.9925	0.9909	0.9926

	*P_val_*	0.9939	0.9907	0.9900

	*ΔP_fit_*	**0.0014**	**-0.0002**	**-0.0026**

to SS model	*P_ver_*	0.9825	0.9986	0.9955

	*P_val_*	0.6769	0.8822	0.6847

	*ΔP_fit_*	**-0.3056**	**-0.1164**	**-0.3108**

to GRLOT model	*P_ver_*	0.9737	0.9672	0.9975

	*P_val_*	0.9712	0.9333	0.9970

	*ΔP_fit_*	**-0.0025**	**-0.0339**	**-0.0005**

Detailed data				

to ANN model	*P_ver_*	0.9999	0.9846	0.9975

	*P_val_*	0.9998	0.9789	0.9967

	*ΔP_fit_*	**-0.0001**	**-0.0057**	**-0.0008**

to SS model	*P_ver_*	0.9916	0.9999	0.9981

	*P_val_*	0.8294	0.9917	0.8321

	*ΔP_fit_*	**-0.1622**	**-0.0082**	**-0.1660**

to GRLOT model	*P_ver_*	0.9936	0.9934	0.9986

	*P_val_*	0.9935	0.9879	0.9982

	*ΔP_fit_*	**0.0001**	**-0.0055**	**-0.0004**

Data (created by ANN, SS, GRLOT methods) predicted by the different methods (for cases A, B, C) and subsequently used for making in silico predictions; for *sparse *and *detailed *data sets.

**Table 5:** Parameter matrix characteristics

	Case A			Case B			Case C		
**Data generation method**	**ANN**	**SS**	**GRLOT**	**ANN**	**SS**	**GRLOT**	**ANN**	**SS**	**GRLOT**

Uniform degradation rate	x - -	x x x	x x x	x - -	x x x	x x x	x x x	x x x	x x x
Constant signal propagation	x - -	- x x	- x x						
Asymmetric signal branching				x x x	- x x	x x x	x - -	- x x	- x x
Asymmetric co-regulation				- - -	- x -	- - -	- - -	x x -	- - -
Negative feedback							x x x	x x x	x x x
Positive feedback							x x -	x x x	x x x

Uniform degradation rate	x - -	x x x	x x x	x x x	x x x	x x x	x x x	x x x	x x x
Constant signal propagation	x - -	- x x	- x x						
Asymmetric signal branching				x x x	- x x	x x x	x - -	x x -	x x x
Asymmetric co-regulation				x - x	x x x	x - x	x - -	x x -	x x x
Negative feedback							x x x	x x x	x x x
Positive feedback							x x x	x x x	x x x

Characteristics found in the parameter matrices of the reverse-engineered network models: Characteristics corresponding to the *sparse *data sets are depicted in the top rows of the table, those corresponding to the *detailed *data sets in the bottom rows. The symbol "x" shows that the feature was discovered by the method, "-" its was not discovered, and spaces indicate that the feature was not part of the case study. The reverse-engineering methods applied to each data set are in the order ANN, SS, GRLOT.

#### Step 5: Validation of reverse-engineered GRN models

*Input*:

• 54 of the 60 GRN models from Step 3 corresponding to the models (A, B, and C) derived from nine sparse and nine detailed data sets using ANN, SS and GRLOT, respectively. Plus 9 reference GRN models, constructed in Step 1, corresponding to three ANN, three SS, and three GRLOT models, respectively.

• The 6 remaining GRN models from Step 3, corresponding to three for each of model D and E.

• The 3 switch-on models from Step 3 for model F.

**Figure 4:**
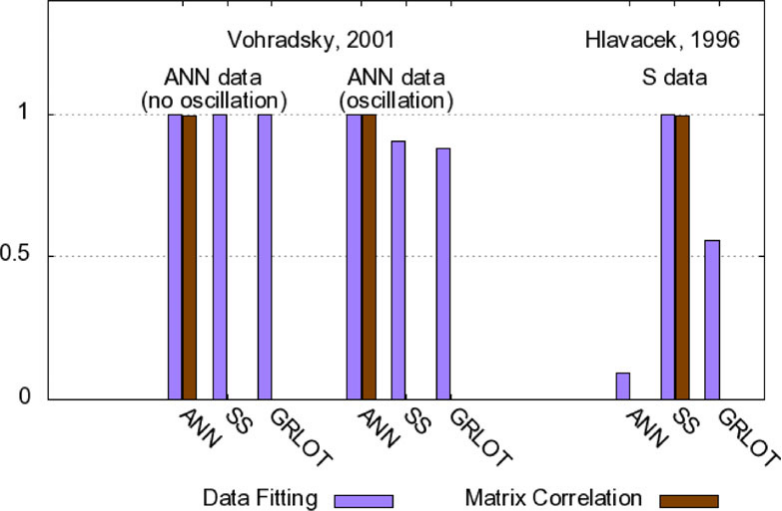
**Data fitting and matrix correlation values for Cases D and E**. Data fitting (predictive power, *P_ver_*, measured on training data) and matrix correlation (i.e., inferential power, *P_inf _*) values for reverse-engineered models for Cases D and E.

*Output*:

• 54 *P_val _*values indicating how well the reverse-engineered dynamic GRN models function under new conditions (i.e., reproduce unseen data). The results of the validation experiments with the reverse-engineered dynamic models are summarized in Table 3 and Table 4, respectively.

• By reproducing data sets based on the 5-gene Hlavacek model (model E) described with the SS method, we analyzed if the ANN and GRLOT methods can be used for reverse-engineering these dynamics without supporting regulation of the degradation process. Accordingly, we analyzed the Vohradsky model (model D) to see if small variations of the external input parameter *ϑ_i_*, which lead to large effects in ANN models, could be reverse-engineered into models based on the SS and GRLOT method. These results are summarized in Figure 4.

• Table 6 describing the results of perturbation experiments performed on the 3 switch-on data sets of model F.

**Table 6:** Perturbation data for the models generated for the Cantone model F

		Comp. to unperturbed network			Comp. to perturbed network		
**Perturbation**		**ANN**	**GRLOT**	**SS**	**ANN to GRLOT**	**ANN to SS**	**GRLOT to SS**

CBF1	0.0045	1	1	1	0.951	0.461	0.337
	0.0135	0.656	0.556	0.556	0.949	0.431	0.345
	0.054	0.059	0.0397	0.0397	0.795	0.613	0.808
	0.216	0.0035	0.0022	-	0.0072	-	-

GAL4	0.0324	1	1	1	0.951	0.461	0.337
	0.0972	0.940	0.549	-	0.542	-	-
	0.3888	0.059	0.0395	-	0.0606	-	-
	1.5552	0.00025	0.0023	-	0.0052	-	-

SWI5	0.0075	1	1	1	0.951	0.461	0.337
	0.0225	0.992	0.994	0.893	0.952	0.310	0.222
	0.09	0.800	0.838	0.217	0.953	0.0672	0.0506
	0.36	0.180	0.221	-	0.953	-	-

GAL80	0.0221	1	1	1	0.951	0.461	0.337
	0.0663	0.541	0.498	0.371	0.930	0.555	0.545
	0.2652	0.0208	0.0354	0.0192	0.915	0.581	0.545
	1.0608	2.09e-05	0.0021	-	0.0022	-	-

ASH1	0.012	1	1	1	0.951	0.461	0.337
	0.036	0.939	0.935	0.780	0.951	0.471	0.342
	0.144	0.338	0.328	0.212	0.950	0.476	0.344
	0.576	0.0272	0.0263	0.037	0.945	0.471	0.338

Perturbation data for the models generated for the Cantone model F [19], based on the switch-on network only. The left hand set of results compares the perturbed network to the unperturbed network: they give an indication of how the perturbation has changed the network dynamics. The right hand set of results compares the perturbed networks across the three methods. The closer the values are to 1.0, the more similar the dynamics. The perturbations involve increasing the initial gene values by ×3, ×12, and by ×48.

### 2.2 Discussion

In order to ensure that our results based on the artificial data sets are fully applicable to biological systems, we first discuss the results from reverse-engineering the biological data (model F). We then discuss scenarios where the *same *methods were used for both reverse-engineering and data set creation: this allows us to check if the methods are able to accurately discover the original parameter matrices used to generate the data and to see if the discovery of the parameter matrices is a necessary condition to generate accurate and realistic dynamics. Next we highlight idiosyncrasies between the methods by discussing the results when *different *methods were used for reverse-engineering and data set creation, and before concluding our discussion by considering specific features of the network models, we describe the results of our qualitative comparisons.

#### 2.2.1 Reverse-engineering biological data

**Figure 5:**
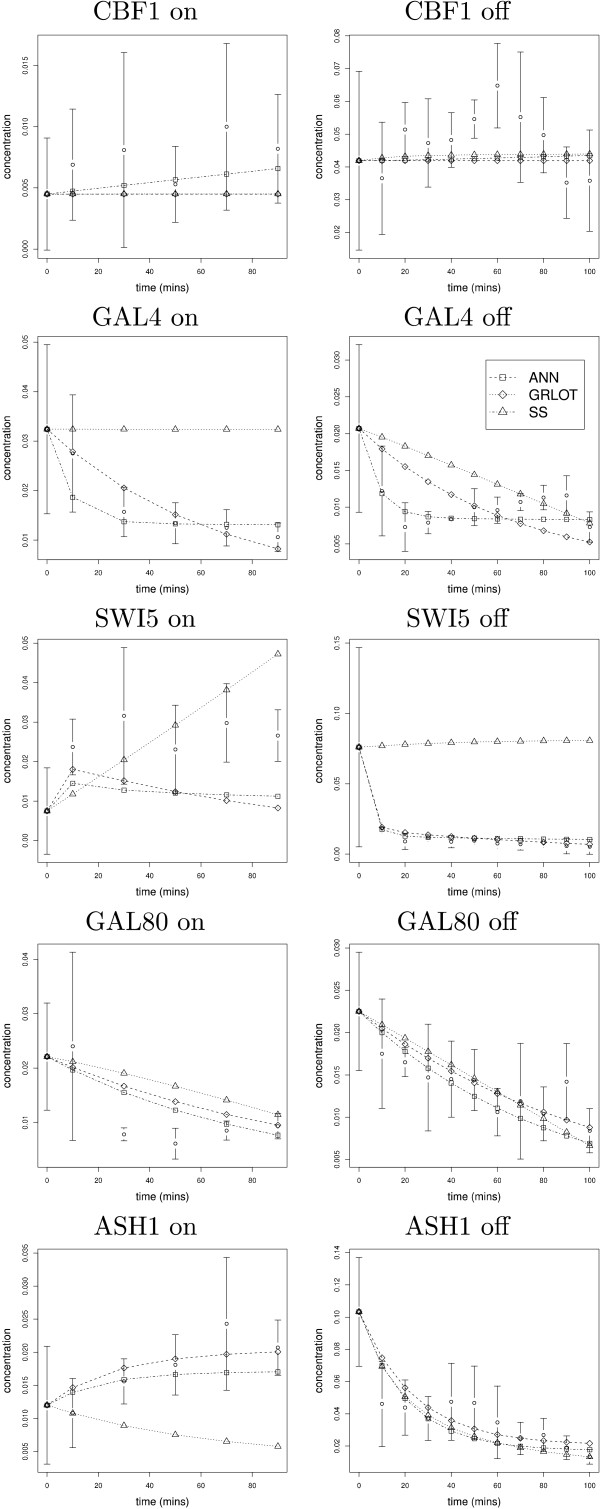
**Switch-on and switch-off dynamics of GRN model F**. Based on the synthetic network and corresponding data sets from Cantone et al. [19] we show the experimentally determined data with error bars for a switch-on and switch-off times-series, along with the modelled gene expression curves generated by each of the three methods.

The ten graphs in Figure 5 show the gene expression data of the five genes in the model F, *CBF1, GAL4, SWI5, GAL80, ASH1 *[19], along with the dynamics predicted by the three methods. The ANN and GRLOT methods tend to provide a good match to the experimental data, with the ANN method giving the best results in this test. The SS is clearly worse than the other two methods due to the sensitivity of its many multiplied power terms. We have found that many of the terms automatically generated by the evolutionary algorithm for the SS method do not give any solution (i.e the integrator fails), and so the method therefore tends to converge on local minima where many, if not all, of the power terms are set to zero, thus resulting in simple linear graphs. However, sometimes a linear solution may be a reasonably accurate approximation to genes that are in a steady-state of expression, and so linear solutions may not have a significantly detrimental effect on the overall model output.

Table 6 summarizes the results for the perturbation experiments performed on the reverse-engineered models. The ANN and GRLOT are again in more accord with each other than they are with the SS method, and the SS method fails more frequently. Perturbing different genes influences the network dynamics in different ways, some giving a large change to the overall network dynamics, and some leaving them relatively unchanged. This is shown in the table by the comparing the perturbed network dynamics to the unperturbed state. When comparing the predicted network dynamics for the different methods, the results indicate that good agreement is possible between the ANN and GRLOT methods, especially for the smallest perturbations, but also sometimes even for the largest perturbations we explored: however, this is not always the case as there was little agreement between the methods when the *GAL4 *gene was perturbed. We believe that these results show that the mathematical methods are able to generate reasonable solutions for biological data sets. In subsequent sections we further elucidate characteristics of the methods using our more detailed artificial data sets.

#### 2.2.2 Within-method estimation of model parameters

We examined the accuracy of model parameter estimation for the situation where the reference GRN model (constructed in Step 1) and the reverse-engineered GRN model are based on the same modeling method (ANN, SS or GRLOT). To assess how accurately the original parameter matrices were discovered, we used the inferential power measure, *P_inf _*, defined by Equation 13.

Table 3 depicts the results we obtained from estimating within-method model parameters of the reverse-engineered models. First, we observe that although the ANN and GRLOT methods achieved relatively low *P_inf _*scores (compared to the SS method), they still performed relatively well in terms of predicting the training data measured by the *P_ver _*scores. The GRLOT method is particularly notable here: For case C on the sparse data set, even though the *P_inf _*value is only 0.6046, the *P_ver _*value is 0.9999, indicating very accurate replication of the model dynamics even with significant errors in the parameter matrices. Indeed, this model also makes highly accurate predictions when it is perturbed by increasing and decreasing inputs. This is in marked contrast to models based on the SS method, which performed relatively well at discovering the original parameters (high *P_inf _*), but made poor predictions (low *P_val_*) when the model was perturbed. This indicates a lack of robustness of the SS method due to its extreme sensitivity. Compared to the SS method, models based on both the ANN and GRLOT methods are much more robust to variations in their model parameters and are thus able to make more accurate predictions (higher *P_val_*) of model behavior.

The experiments with the detailed data sets confirmed these results. As expected, all methods perform better with the more detailed data set (providing more training data reflecting different initial conditions), and even the SS method improved the quality of its predictions, but it was still outperformed by the ANN and GRLOT methods.

#### 2.2.3 Reverse-engineering different method data

Looking at the situation when the methods reconstructed (predicted) all data sets generated by all the methods, we relied on the *P_ver _*measure as the methods were unable to reverse-engineer the original parameter matrices in all cases. The results of these experiments are summarized in Table 4.

As expected, the quality of the models is lower when the methods predicted the data created by one of the other methods. The SS method is conspicuous in its poor performance. In Table 3, the *P_inf _*values for the SS method indicate that the parameter matrix was discovered with very high precision (the values are 0.9994 for sparse data and 0.9999 for detailed data). However, the Δ*P_fit _*values, which indicate the performance of the models when making predictions about network behavior under changes in input, are very poor: the values for cases B and C are 0.1358 and -0.2095, respectively, for the sparse data and 0.0118 and 0.0109, respectively, for the detailed data. Bearing in mind that a Δ*P_fit _*value of 0.1000 or above is of poor quality, with almost no visible relation to the original or expected dynamics, and that accurate models would require a Δ*P_fit _*value of about 0.0100 or less (see in Section 4.5.1), it then becomes clear that the ability of the SS method to make satisfactory predictions about network behavior is much less than that of the ANN and GRLOT methods, even when highly sampled data of near-perfect accuracy is used (i.e., artificial data created by the SS method). This inaccuracy is due to the exponential terms in the SS method, which are highly sensitive to its parameters even when they are accurate to three or four decimal places. A decrease in model input corresponds to an increase in the degradation rate *k_1 _*(or *β_1_*) on gene *X_1_*, as described in Section 4.4. Due to the nature of the dynamics, the *k*_1 _increase is a smaller perturbation than the decrease, corresponding to the differences in these average values for *k*_1 _= 0.5000 and *k*_1 _= 0.1000, respectively. The individual data for input increase and decrease predictions show that the SS method actually performed as well as the other two models when the input was decreased, the problems arose when the input was increased. The results therefore indicate that the *SS models may be reasonably robust to small perturbations, but are highly sensitive to larger perturbations and consequently liable to lose all predictive ability*.

It is also worth noting that the SS method performed poorer in reverse-engineering well-performing GRN models based on data generated by the GRLOT and ANN methods than the GRLOT and ANN methods in reverse-engineering models based on the data created by the SS method. The ANN and GRLOT also performed slightly worse when reverse-engineering from SS data than when reverse-engineering from data created by the GRLOT or ANN method, respectively.

#### 2.2.4 Qualitative comparison of network features

The results of the qualitative comparison between the methods, as described in Section 4.5.3, are summarized in Table 5. They show that the GRN models reverse-engineered from the detailed data sets had a higher similarity to the original models, in terms of their underlying network features, than models based on the sparse data set. This result portrays the opposite of what is observed on the basis of *P_ver _*values, where the reverse-engineered models score higher with the sparse data than the detailed data sets. This result is an indication of the problems that arise with discovering the underlying network features when the data availability is low.

In the following we consider different network features individually:

##### Uniform degradation rate

With reference to sparse data, Table 5 (block of top rows), the results show that the ANN method was highly successful in discovering uniform degradation rates in each of the three cases and with each data set. However, the other two methods performed better in predicting data generated by the SS or GRLOT method than by the ANN method. Even with detailed data, the SS and GRLOT methods failed to discover the uniform degradation rate as simulated by the ANN method, especially with case A, although all methods discovered this feature when generated by the SS and GRLOT methods.

##### Constant signal propagation

Only case A has the constant signal propagation feature. There is a clear difference between the methods: The ANN method was able to reproduce its own data, but not that of the SS or GRLOT method, whereas the SS and GRLOT could reproduce their own and each other's data, but not the data generated by the ANN method.

These findings can be explained by the different way in which the three methods process inputs. For example, constant signal propagation from one gene to another described with uniformly weighted input values (as realized by the ANN method) can certainly not be reproduced with constant signal propagation defined with uniformly exponentiated input values (SS method).

##### Asymmetry in signal branching and co-regulation

Asymmetric signal branching and co-regulation are features in the case studies B and C. For sparse data, the methods were less successful at discovering asymmetric co-regulation than asymmetric signal branching, but for detailed data both features were modeled with similar success rates. As with the case of constant signal propagation, the different approaches to input processing of the three methods seem to explain this observation.

For sparse data, the asymmetric co-regulation feature was not discovered at all when reverse-engineering from data generated by the ANN and GRLOT method, respectively. In case B, co-regulation involves the interplay between the two genes, X3 and X4, that control the expression of gene X5. By looking into the details of the models (data not shown here), the ANN and GRLOT models either described co-regulation with interchanged asymmetry or contain, incorrectly, an inhibitory dependency of gene X5. The sparse data set was not sufficient to ensure that this characteristic was reflected in the reverse-engineered models, but for detailed data the methods were more successful and in particular the ANN method could correctly reproduce co-regulation for all the data sets.

##### Positive and negative feedback loops

These features are only found in the static GRN of case C. The methods have performed relatively well in reverse-engineering these features. Specifically, all models reverse-engineered from one time-series (Table 5, block of rows at the top) contain the positive and the negative feedback loops of network case C, with the exception of one GRLOT model that was reverse-engineered from ANN data. This GRLOT model discovered an extra negative feedback loop instead of the positive feedback loop.

All models reverse-engineered from the detailed data rediscovered the feedback loops. Feedback loops play a key role in the model dynamics of network case C, and are therefore essentially contained in nearly all reverse-engineered models.

#### 2.2.5 Considering the different case studies

This section analyzes the different case studies in some detail.

##### Cases A, B, and C

Generally, the increasing complexity of the static GRN models from A to C had no significant impact on the predictive power of the reverse-engineered models on the training data (*P_ver _*values). This, however, might be different in more complex reverse-engineering settings where the network topologies are not known.

##### The Vohradsky case D: Oscillatory dynamics

While models based on all three methods were able to obtain high *P_ver _*values on reproduced *non-*oscillatory dynamics, the SS and GRLOT models reverse-engineered from the time series did not perform so well for *oscillatory *dynamics. The data sets used for these experiments are shown in the left and right panels of Figure 3.

**Figure 6:**
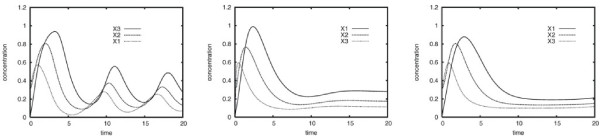
**The oscillatory dynamics of three models**. The oscillatory dynamics of the ANN model (left), cannot be reproduced in the SS and GRLOT models (middle) and (right).

The reason for the ANN method's ability to model oscillations lies in the external input parameters *ϑ_i _*which can be used in ANN models to modulate the responsiveness of each gene to incoming signals individually. The ANN model used to generate the non-oscillatory dynamics used identical *ϑ_i _*values whereas the underlying model of the oscillatory dynamics used different *ϑ_i _*values to define fast and delayed signal responses. As shown in Figure 3, this led to oscillatory dynamics caused by slower responses within the cyclic negative feedback system defined in the Vohradsky scenario. These oscillations could not be reproduced by the reverse-engineered SS and GRLOT models (see Figure 6). Thus, the oscillatory dynamics is extremely biased towards the individual characteristics of the ANN method and are therefore difficult to model by dynamic GRN models based on the SS or GRLOT methods.

To better understand the data fitting verification method (based on the training data), it is useful to compare the time series shown in Figure 6 with the *P_ver _*values given in Figure 4: the *P_ver _*values are still relatively high for the SS and GRLOT methods, despite their failure to reproduce the oscillatory dynamics seen in the left diagram of Figure 6.

##### The Hlavacek case E: singularities

The same conclusions can be drawn from the results obtained with the Hlavacek case. The SS time series used here was also strongly influenced by unique characteristics of the SS method. First, the underlying network model of the test data set [38] involves the regulation of the degradation process of X3. More importantly, though, is the fact that singularities have a significant impact on the dynamics generated with this test model. As the *P_ver _*values of Figure 4 show, this time series could therefore only be reproduced by the original SS method, whereas the ANN and GRLOT methods failed.

## 3 Conclusions

In this study we examined three systems of ordinary differential equations commonly applied in the modeling and reverse-engineering of GRNs. Our goal was to assess how the characteristics of the individual mathematical methods affect the quality of the reverse-engineered dynamic GRN models. Our results suggest that the methods are clearly not equivalent and interchangeable and that there are considerable differences in the way the methods process inputs. In particular, the SS method suffers from a relatively high degree of sensitivity to its parameters due to multiple input processing based on multiplied unrestricted exponentials. Thus, for the SS method, even when the original parameter matrix used to generate artificial test data sets was discovered with high accuracy the method sometimes failed to adequately reproduce the network dynamics.

Different methods seem to be suitable for modeling different types of dynamics or network features. For example, oscillatory dynamics may be modeled with the ANN method by using different values within its parameters. However, this may not possible with the SS and GRLOT models, which do not provide straightforward mechanisms for modeling oscillations.

Models reverse-engineered from under-sampled data, based on only a single experiment, are unlikely to discover the real parameter matrix, and should be regarded with considerable caution. Even when multiple, well-sampled time series data are available, it is does not seem wise to rely on models based on just a single modeling method, as it is likely to have some bias. The SS method, in particular, is prone to generating unrealistic dynamics, especially for relatively large input perturbations where our experiments showed its predicted output to be very inaccurate (see Section2.2.2). Even for very small gene networks, considerable amounts of highly time-resolved (many sampling points) and highly condition-resolved (based on multiple perturbations) data may be necessary to produce reliable dynamic GRN models. Furthermore, as the complexity (number of genes and regulatory interactions) increases, the computational complexity (compute power) required to reverse-engineer dynamic GRN models becomes non-trivial, and may require non-standard computing solutions such as clusters, supercomputers or other large-scale computing solutions.

In the experiments we performed, SS-based dynamic GRN models generally yielded significantly less accurate predictions than the ANN and GRLOT methods. While some features such as positive and negative feedback loops were correctly modeled in almost all situations we studied, it was often the case, especially when using the sparse data sets, that the methods failed to reverse-engineer features such as uniform degradation rates, constant signal propagation, asymmetry in signal branching and asymmetry in co-regulation. Even with the detailed data sets, these features could not be consistently reverse-engineered. Of the three methods, perhaps the ANN method deserves more attention due to its reliability and the relatively small number of parameters it requires. Indeed, although in this paper we have not presented results for the amount of computation required for each method, it is important to note that if the amount of computational power is limited, then the ANN method generally produces the most accurate and robust models for the least computation.

## 4 Methods

Before we describe the experimental design we used to compare the GRN modeling methods, we introduce some terminology.

### 4.1 Terminology

#### Static GRN model

The static model of a GRN represents the regulatory interaction *structure *or *topology *of a GRN. It describes whether or not there is a regulatory relationship between genes and what the nature of the interaction is, if it exists. The interaction nature is a binary property. It describes the influence of interacting genes, i.e., whether a gene has an activating (up-regulating, inducing) or inhibiting (down-regulating, repressing) influence on another gene. A static GRN model does not quantify gene expression amounts or regulatory influence. Static GRN models are easy to depict diagrammatically as a graph or network in which a *node *represents a gene and an edge a represents a regulatory interaction between two genes. The type and direction of an interaction is typically depicted symbolically: An arrow head represents activation, a bar represents inhibition. A diagrammatic depiction of the static GRN models used in our study is shown in Figure 2.

#### Dynamic GRN model

A dynamic GRN model refers to a representation that captures both the regulatory interaction structure (including the nature of the interactions) as well as quantitative rules that describe how gene expression amounts vary (in response to perturbations) over time.

#### Dynamic gene expression data

In general, dynamic gene expression data represent the time-variant expression levels (abundance of functional gene products: RNA, proteins) of the genes comprising a GRN. In our study we do not use dynamic gene expression data from real biological experiments. Instead, we generate dynamic gene expression data from dynamic GRN models with manually defined model parameters and (initial) expression levels.

#### Reverse-engineering of GRNs

Reverse-engineering of a GRN refers to the process of constructing (by automated means) a dynamic GRN model based on dynamic gene expression data. In particular, the reverse-engineering of a dynamic GRN model requires the reconstruction (from data) of the underlying regulatory interaction structure as well as the parameters governing the quantitative regulatory influence among genes. We simplified the reverse-engineering problem somewhat by avoiding the reverse-engineering of regulatory interactions that are not present in the static GRN models used as case studies (Figure 2).

### 4.2 Model simulation: Generating the training data sets

For the reference GRN models obtained from the static GRN models A, B and C, the initial concentration levels of the model variables *X_i _*are identical throughout all models and comprise five sets (called *starting sets*) leading to different dynamics (see Table 7). In the starting set 1, for instance, the high initial concentration of X1 triggers the dynamics of the other network components, whereas the concentrations of starting set 5 are more evenly distributed.

**Table 7:** Initial model inputs

	X1	X2	X3	X4	X5
Starting set 1	0.8	0.1	0.1	0.1	0.1

Starting set 2	0.8	0.1	0.1	0.3	0.5

Starting set 3	0.5	0.5	0.2	0.1	0.1

Starting set 4	0.4	0.8	0.2	0.2	0.2

Starting set 5	0.5	0.2	0.8	0.8	0.2

Initial mRNA concentration levels chosen for all models. We generate a sparse data set comprising one time-series based on starting set 1 and a detailed data set which comprises five time-series based on starting set 1 to starting set 5.

The dynamics generated by the different modeling methods for the same GRN differ considerably. There are two main reasons for this: First, we chose model parameters producing different dynamics to provide a variety of expression profiles to facilitate the comparison of the three modeling methods. Here, the idea is to assess to what extent the *reverse-engineered *dynamic GRN models are able to reproduce the dynamics generated by the reference models. This relates to the model verification Step 4 in our study design. Second, the fact that the different reference models generate different dynamics is also explained by idiosyncratic characteristics of the modeling methods.

With the dynamic GRN reference models constructed from the static GRN models A, B and C the following dynamic gene expression data sets were generated (see Step 2 in our study design in Figure 1):

1. *Sparse *data sets, comprising the dynamic gene product concentrations of all genes but based on only a single set of initial conditions, namely starting set 1 (see Table 7).

2. *Detailed *data sets, based on five sets of initial conditions, namely based on starting set 1 to starting set 5.

3. All time series consist of 200 data points, each data point describing the gene product concentration *X_i_*(*t_k_*) at time point *t_k _*for each gene *i *= 1, ..., *n*.

The model parameters for the reference models of the Vohradsky model (case D), the Hlavacek model (case E), and the Cantone model (case F) were taken directly from the literature [15,19,38].

### 4.3 Reverse-engineering of dynamic GRN models from time-series data

Many approaches to reverse-engineering of GRNs exist [12]. In our study reverse-engineering came down to a parameter estimation or optimization problem (Step 3 in our study design). We adopted an evolutionary algorithm approach to address this task [20]. Different reverse-engineered dynamic GRN models may be generated with different optimization techniques. However, we are confident that the parameter optimization technique we adopted was successful because, for the ANN and SS methods, we could demonstrate that the parameters of the reference models could be reverse-engineered with high accuracy (see the *P_inf _*values in Table 3). Furthermore, the validation results (predictive power on unseen data) for some of the dynamic GRN models we reverse-engineered with the evolutionary algorithm technique, show high levels of accuracy. Details of the optimization approach are discussed below.

#### 4.3.1 The parameter optimization problem

The primary purpose of dynamic GRN models is to reveal regulatory mechanisms of biological gene regulation networks. Typically, in the case of highly abstracted GRN models as discussed here, dynamic (time-variant or time-series) gene expression data forms the input to the reverse-engineering process. When reverse-engineering dynamic GRN models from experimental time-series data, the problem is to identify model parameters for which the discrepancy between the experimental data and the data predicted by the model is minimal. This leads to an optimization problem in which the following function is to be minimized.

(5) $$f=\sqrt{\sum_{t=1}^{T}\sum_{k=1}^{N}{\left(X_{k}(t)-{\hat{X}}_{k}(t)\right)}^{2}}$$

where

*X_k_*(*t*) denotes the experimentally *observed *gene product concentration level of gene *k *at time point *t*.

${\hat{X}}_{k}(t)$ denotes the gene product concentration level of gene *k *at time point *t predicted *by the dynamic GRN model.

*N *denotes the number of genes in the network and *T *the number of sampling points of observed data.

This approach is based on the assumption that the minimum of function *f *corresponds to the set of parameters (i.e., parameter matrix) of the dynamic GRN model for which the model's outputs optimally fit the experimental data [28].

We performed optimization using evolutionary algorithms [20,39-41], which are stochastic search methods that mimic, on a highly abstracted level, biological evolution and sexual reproduction. Each evolutionary algorithm works with a population of points on the problem space where points are represented by *individuals*. Transformations on the individuals will tend to assemble individuals in more favorable areas of the search space, i.e., areas with high evolutionary *fitness*. When all or most individuals are located within the same area then a local (and possibly global) optimum of the search space is found.

Our approach was realized as follows. Individuals are represented by real-valued vectors, and each value can evolve within a defined variable-dependent interval. Here, the different genetic operators acting on those vectors ensure that the values remain with the specified intervals. First, we generated a random initial set or population of individuals. In this case, individuals are generated by setting random values to the unknown model parameters of the differential equations representing the dynamic GRN models (with the three modeling formalisms ANN, SS and GRLOT). A simulation was then performed for each individual and this was used to evaluate the fitness by comparing how well the simulated model output matches the time-series expression data (see Equation 5). The individuals were then ranked according to their fitness and in the next step we selected individuals for genetic operations, including *cloning, recombination *and *mutation*. These processes of selection, recombination and evaluation were repeated until a designated termination criterion was met, such as the attainment of an acceptable fitness level, or if a certain number of generations were evaluated.

#### 4.3.2 The two-phase reverse-engineering strategy

In this study, we applied a two-phase strategy to reverse-engineer dynamic GRN models from time-series gene expression data created in Step 2 from the reference models. We refer to the first phase as *bottom-up phase *and the second as *top-down phase*. We also employed the notions of a *network model*, denoted by **m**, and a *node model*, denoted by *m_k_*, to structure reverse-engineering problem [42].

**Figure 7:**
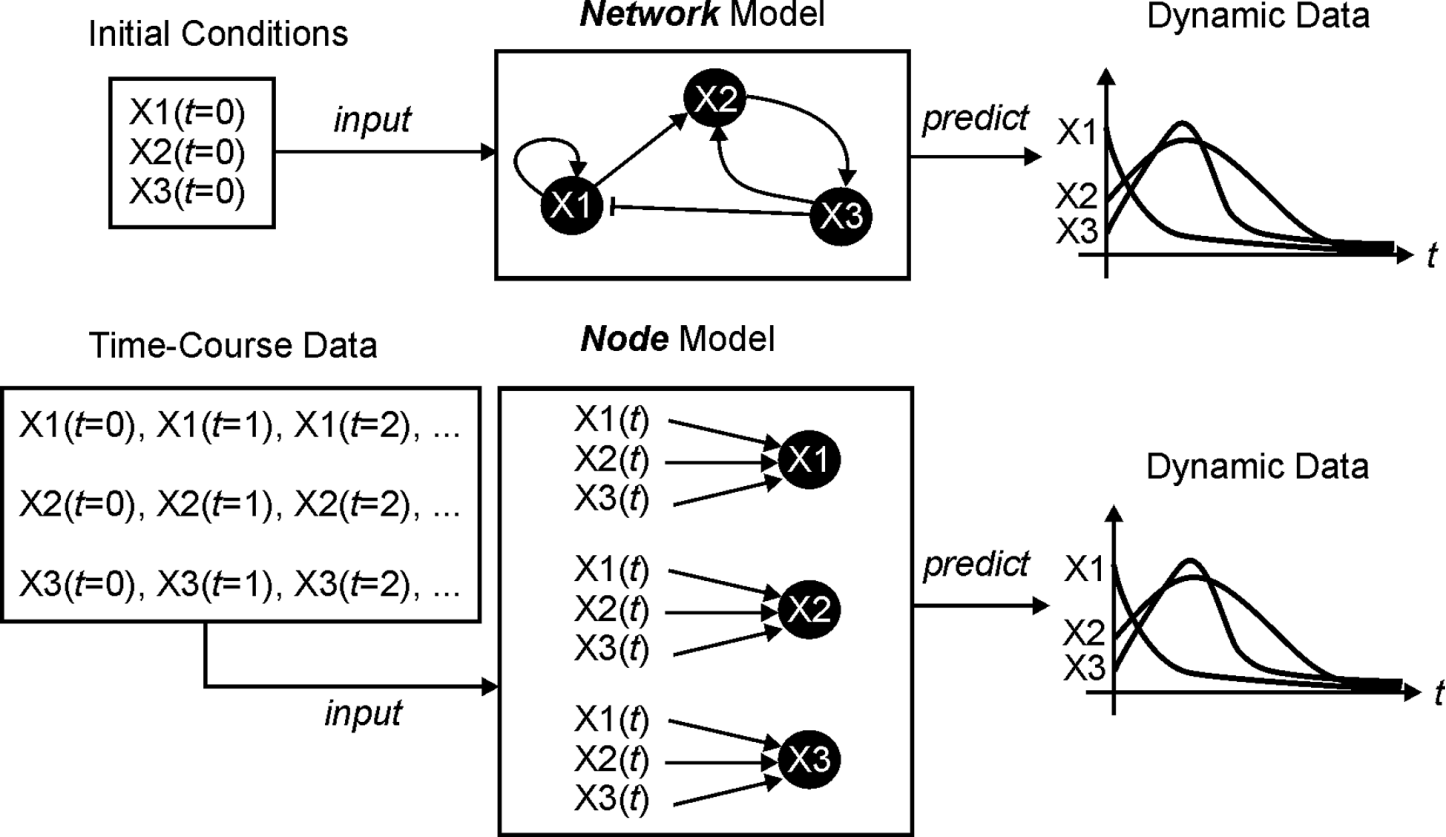
**Creating molecular and network models**. The process describing the generation of GRN models from *molecular models*.

Figure 7 illustrates the concepts of the node and network models. The node model describes the regulatory dependencies of a single gene, whereas the network models contain the dependencies of all genes of a network. Both types of model can be simulated: A network model requires only the *initial conditions *of constituent elements (i.e., gene product concentrations). In contrast, the node model requires as input the the time-dependent levels of all the elements. Consequently, node models usually fit the experiment very accurately, since they can use the experimental data as input, yet they are unable to predict behavior of the system under new experimental conditions.

The number of parameters to estimate in all three modeling methods ANN, SS and GRLOT increases with *O*(*N*^2^), where *N *is the number of genes in the GRN. This makes it very difficult to reverse-engineer large-scale GRN models. Recently, a variation on the "network" model of reverse-engineering mentioned here was further developed based on the ANN method by Vohradsky and colleagues [43,44]. Even the reverse-engineering of relatively small GRNs, as presented by this study, turns out to be challenging. The advantage of node models, however, is that they require only $\frac{1}{N}th$ of the parameters required for the network model consisting of *N *genes. Thus, a considerable complexity reduction can be achieved by decomposing the parameter optimization problem of a GRN into the smaller problem of *N *node models with complexity *O*(*N*). This problem decomposition approach was proposed as a *step-by-step strategy *by Maki and colleagues [45] and successfully applied elsewhere [27,33,46].

Formally, we define a *network model*, **m**, as a structure comprising *N *node models, *m_k _*as follows:

(6) $$m=(m_{1},m_{2},m_{3},\;\ldots ,m_{N})\;k=1,\ldots ,N.$$

Such a network model, **m**, is capable of *predicting *the dynamics of a GRN consisting of *N *genes. Each constituent node model, *m_k _*of **m**, approximates the concentration changes, $d{\hat{X}}_{k}/dt$, captured by the experimental data according to the following function:

(7) $$\frac{d{\hat{X}}_{k}}{dt}=f(X(t),\;w_{k}),$$

where

$\frac{d{\hat{X}}_{k}}{dt}$ denotes the approximated gene product concentration changes of gene *k*,

**X**(*t*) denotes the concentration levels of observed gene product concentrations at time *t *as provided by the dynamic gene expression data,

**w**_*k *_is a vector representing the parameters of the node model *m_k_*.

To approximate the concentration dynamics of a gene within an entire *network model*, we define accordingly:

(8) $$\frac{d{\hat{X}}_{k}}{dt}=f(X(0),\hat{X}(t)w_{k})$$

Here $\hat{X}(t)$ describes the approximated concentration levels of the nodes (representing gene products) *k *in the network and **X**(0) specifies the initial condition or state of the network model (as obtained from the time-series data).

In the bottom-up phase of our two-stage approach, we estimated the parameters ***w***_***k ***_of each individual node model, *m_k_*, independent from the parameters of other node models. Subsequently, in the top-down stage, we reverse-engineered (optimized) the parameters, **W**, of the entire network model, **m**, by taking the results of the bottom-up approach as a starting point.

##### Bottom-up phase

In the bottom-up phase the parameter estimation problem for one network model, **m**, is divided into smaller parameter estimation problems for *N *node models, *m_k_*. The parameters, ***w***_***k***_, of the *N *molecular models can be reverse-engineered step-by-step and independently from each other. This is facilitated by the extraction of the concentration levels, *X_i_*, from all genes in the experimental data set, **D**. Hence, the objective function for reverse-engineering optimal node models from experimental data can be reduced to

(9) $$f_{k}=\sqrt{\sum_{t=1}^{p}{\left(X_{kt}-{\hat{X}}_{kt}\right)}^{2}}$$

This function corresponds to the fitness of the *k*-th node model where ${\hat{X}}_{kt}$ is the calculated gene expression level of gene *k *and *X_kt _*is the given expression level of gene *k *at time *t *from the data set **D**.

With several repeated iterations of the bottom-up process, the intention was to find one or more optimal parameter sets *w*_*k*_*i *which produce small error values according to Equation 9. By combining the fittest of the *N *identified node models form *m_k_*, it is then possible to construct a self-contained network model, **m**, which can simulate the dynamic behavior of the network independently from the gene expression matrix

**D**. Consequently, and in contrast to the node models *m_k_*, a network model, **m**, is capable of simulating or predicting scenarios (gene product concentrations) that are not captured by the experimental data.

##### Top-down phase

In the top-down phase, we estimate parameter sets **W **= (*w*_1_,*w*_2_, ..., *w*_N_) of network models, **m**. Here, the concentration levels $\hat{X}(t)$ and the parameters sets, **w**_k_, of all node network models, *m_k_*, in **m **are estimated together and dependent upon each other. So this case involves the interplay between the individual node networks in that the estimated concentration levels of each node network provide the potential input for other node models. In this case, the objective function for reverse-engineering optimal network models from experimental data is defined as

(10) $$f=\sqrt{\sum_{t=1}^{p}\sum_{k=1}^{N}{\left(X_{kt}-{\hat{X}}_{kt}\right)}^{2}}$$

As already discussed, it is possible to construct a network model, **m**, by merging of node models, *m_k_*, found in the bottom-up phase. At this stage, however, the network model has not learned to work as a system that is aware of the interplay among the individual node models. Since the network model no longer derives its input from a static gene expression data matrix, **D**, but instead is estimating the concentration levels for each network node, it can show dynamics totally different from the dynamics generated by the individual node models. The reason for this discrepancy lies in error propagation, where even small variations at the beginning of a simulation can cause dramatic fluctuations at later stages. For this reason, a network model constructed from node models is not the final result but rather constitutes a good starting position for the global parameter optimization.

#### 4.3.3 The parameter sets used

To identify optimal parameter sets for the different gene expression models, we applied an evolutionary algorithm in which the genotype is directly based on real-valued vectors [41]. The genetic operators applied in our study are *BLX-α crossover *[47], with a crossover rate of 0.6, and *real number creep mutation*, with a mutation rate of 0.1. The selection method used for selecting individuals for the generation changes is roulette-wheel selection with proportionality to the positions found in a rank-based fitness assignment operation. The number of individuals contained in one population was 500 and the number of generations was 300. Each parameter optimization for node models and for network models was repeated five times. This reverse-engineering strategy was able to precisely reproduce (often to more than 4 decimal places) the original parameter matrices for situations where the modeling method of reference model (Step 1) and reverse-engineered model (Step 3) were identical.

For consistency, we used the exact same approach (i.e., the same evolutionary algorithm parameters such as population size, number of generations, etc.) for all the reverse-engineering experiments. The only exception to this was the Cantone model F that we worked on at a time when our usual infrastructure, described in Section 4.6, was not available. Instead we used a similar evolutionary algorithm on the Grid'5000 infrastructure [48].

The parameter space searched for the three methods was defined as follows:

• The ANN method: *w_ij _*from -15.0 to 15.0, *v*_i _from 0.0 to 3.0, *ϑ_i _*from 0.0 to 7.0, and *k_i _*from 0.0 to 2.0.

• The SS method: *g_ij _*and *h_ij _*from -3.0 to 3.0, and *α_i _*and *β_i _*from 0.0 to 15.0.

• The GRLOT method: *I_j _*, from -5.0 to 5.0, *A_k _*from 0.0 to 7.0, *Ki_j _*and *Ka_k _*from 0.0 to 40.0, and *k_i _*from 0.0 to 2.0.

### 4.4 Model validation (Step 5)

Finally, we performed a small number of experiments in which we first altered the initial conditions of the manually constructed reference models (Step 1), then simulated these models to generate dynamic gene expression data (serving as unseen validation data), and then investigated how the reverse-engineered dynamic GRN (Step 3) models behaved when identical perturbations were applied.

In all models, we varied the dynamics of gene product X1, by modifying the degradation rate *k*_1 _in the GRLOT and ANN models, and the *β*_1 _value in the SS models. This manipulation could be viewed as a variation of network input or network stimulus, since in all the case study models, X1 did not depend on the products of other genes.

**Figure 8:**
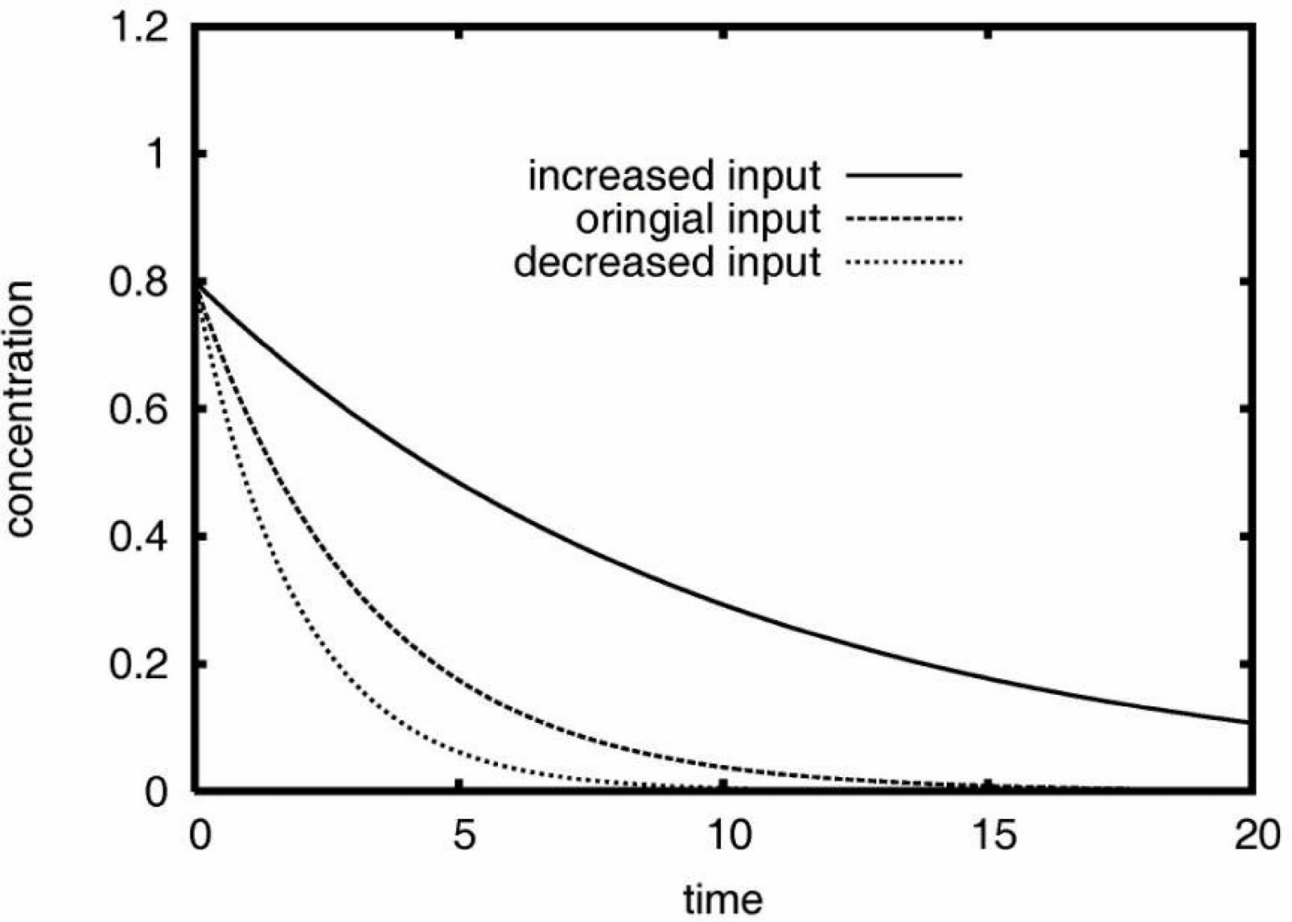
**Dynamics with an input change**. Dynamics of the original gene product concentration of X1 based on a degradation *k*_1 _or *β*_1 _of 0.3. An increased transcript concentration is simulated by decreasing this value to 0.1. Conversely, decreasing transcript concentration levels are simulated by increasing this value to 0.5.

Figure 8 shows the dynamics of the original gene product concentration X1, and an increased and decreased development of gene product concentration levels. The difference of the dynamics, as calculated by using Equation 12, demonstrates the power (predictive power, *P_val_*, on unseen data) of the reverse-engineered dynamic GRN models to reproduce the dynamic data generated by the manually created GRN reference models under different conditions.

For the Cantone model F, the reverse-engineered models were used for perturbation experiments by perturbing the initial value of each gene in turn (whilst keeping the other initial values constant and equal to their respective values for the initial time step in the two time-series data sets). The perturbed value was increased from its initial value by increasing it by ×3, ×12, and by ×48. Equation 11 (which differs slightly from Equation 5) was used to calculate differences between simulated expression models:

(11) $$f=\sqrt{\sum_{t=1}^{p}\sum_{k=1}^{N}{\left(\frac{X_{kt}-{\hat{X}}_{kt}}{X_{kt}}\right)}^{2}}$$

where, ${\hat{X}}_{kt}$ and *X_kt _*are the simulated mRNA levels at time *t *of molecule *k*. *N *is the number of genes in the network and *p *is the number of sampling points of observed data.

### 4.5 Measures to verify and validate the reverse-engineered GRN models

Wessels and co-workers [28] defined measures for assessing the accuracy of reverse-engineered GRN models. Following their approach, we used the following measures (see also Step 4 and 5 of our study design):

• *Predictive power*, *P_ver_*, on training data. This measures how well a reverse-engineered dynamic GRN model reproduces the dynamic gene expression data from which it was generated. So the steps involved are: (a) Create training data from reference models (Step 2); (b) Reverse-engineer dynamic GRN models from training data (Step 3); (c) Execute or simulate reverse-engineered models (resulting in predicted dynamic gene expression data) and calculate *P_ver _*by comparing the training data with the predicted data (Step 4).

• *Predictive power*, *P_val_*, on unseen test data. This measures how well a reverse-engineered dynamic GRN model reproduces *unseen *dynamic gene expression data generated by the reference models under initial conditions different to those used to generate the training data. So the steps involved are: (a) Rerun the reference models (Step 1) under altered initial conditions to generate unseen test data; (b) Apply the same changed initial conditions to the dynamic GRN models generated by Step 3 and re-run the models under these conditions to predict dynamic gene expression data and calculate *P_val _*by comparing the unseen test data with the predicted data (Step 5).

• *Predictive power*, *P_fit_*, for data fitting. We use *P_fit _*when we refer to predictive power in a general way of fitting the data of a predicted data set with another (training or test) data set.

• *Inferential power*, *P_inf _*, measures to what extent the parameters (representing GRN regulatory structure and quantitative influence) of a reverse-engineered dynamic GRN model (Step 3) correspond to the parameters of a reference model (Step 1). We also use terms like *matrix correlation *to refer to *P_inf _*, as the calculation of *P_inf _*involves the comparison of the matrices representing the parameters of the reference (Step 1) and reverse-engineered (Step 3) dynamic GRN models respectively.

• *Qualitative comparison*, *Q_com_*, refers to the extent a reverse-engineered GRN model corresponds with a reference model in terms of certain GRN *features*.

#### 4.5.1 Data fitting and predictive power

Essentially, *P_fit _*compares two sets of dynamic gene expression data, either predicted data with training data (here *P_ver_*, to *verify *a reverse-engineered model), or, predicted data with unseen test data (here *P_val_*, to validate a reverse-engineered model). The general way to compute this score is the same and is defined as follows [28]:

(12) $$P_{fit}=\frac{1}{\left(1+E\right)}$$

where the error

$$E=\frac{1}{pN}\sqrt{\sum_{t=1}^{p}\sum_{k=1}^{N}{\left(X_{kt}-{\hat{X}}_{kt}\right)}^{2}}$$

where *X_kt _*denotes an experimentally observed gene product concentration level at time *t *of gene *k *and ${\hat{X}}_{kt}$ denotes a corresponding gene product concentration level estimated or *predicted *by the dynamic GRN model. *N *denotes the number of genes in the GRN and *p *the number of sampling points of the dynamic gene expression data.

*P_fit _*assumes values form the unit interval, where 1 represents perfect accordance between the experimental data and the simulated or predicted data. However, care needs to be taken when interpreting *P_fit _*values. In Table 8, we indicate the model accuracy depending on the *P_fit _*value. In data generated with our reference models, expression levels usually reach values of no more than 5 units. Thus *P_fit _*values less than 0.9 indicate very poor quality of the reverse-engineered models, with dynamics that bear no relation to the original model. *P_fit _*values between 0.95 and 0.99 indicate models with dynamics in much the same range as the original models, but the expression curves of the individual genes may exhibit different patterns.

**Table 8:** Meaning of *P*_*fit *_values

*P_fit _*value	**Max. RMS diff**.
0.40	244.0

0.75	76.9

0.92	8.47

0.97	2.34

0.992	0.80

0.997	0.42

0.9992	0.13

0.9997	0.067

Interpretation of *P_fit _*(*P_ver _*and *P_val _*) values: The typical maximum *root mean square *(RMS) difference (at a single time point) between the reverse-engineered and reference GRN models, for the 5-gene models of case studies A, B, and C.

*P_fit _*values over 0.99 start to converge with the original model dynamics and a *P_fit _*value of over 0.999 indicates a highly accurate accurate GRN model.

#### 4.5.2 Matrix correlation: Inferential power

*P_inf _*is a deeper measure than *P_fit _*and its variants *P_ver _*and *P_val_*, respectively [28]. It describes more fundamental properties (model *parameters*) of the process that generates *data *which is the basis for calculating *P_fit_*. In our study, we use *P_inf _*to compare the parameter matrix, **W**, of reference models (used to generate the training and test data) with the parameter matrix, $\hat{W}$ of reverse-engineered models.

Inferential power, *P_inf _*, is defined as follows:

(13) $$P_{inf}(W\text{,}\hat{W})=0.5(1+p(W\text{,}\hat{W}\text{))}$$

where *p *denotes the Pearson product-moment correlation coefficient between **W **and $\hat{W}$.

*P_inf _*from Equation 13 assumes values from the unit interval; a value of 1 indicates an exact inference (estimation) of the model parameters. If there is an exact match between the parameter matrices of the reverse-engineered and the reference GRN model, then the behavior of the two networks is identical under all conditions.

Since the three methods ANN, SS and GRLOT use different numbers of parameter values, we can only calculate *P_inf _*values for the cases where the method used for reverse-engineering equals the method used for generating the training data. In all other cases we make a qualitative estimation of *P_inf _*.

#### 4.5.3 Qualitative comparison

*Q_com _*refers to the similarity of a model with the true underlying system in terms of network features.

Primarily, network features refer to the network connectivity that captures the topology of a network and the connection "logic". However, in this study the network structure is given so that only network features such as the type of the effect (inhibitory or stimulatory) and its degree of influence need to be deduced or estimated. Network features are represented by the model's parameters. Since parameters of the three types of mathematical methods can not be directly compared to each other, we determine *Q_com _*by means of a qualitative comparison.

The qualitative analysis of the derived model parameters is performed according to the characteristics listed in Section 1.3.1. For example, when manually constructing the reference GRN models, we chose uniform parameter values to define degradation rates. Based on this and other characteristics described in Section 1.3.1, it is possible to estimate the accuracy of each model.

In this study, the characteristic *uniform degradation rate *is present in the reverse-engineered parameter matrices if all degradation rates within a matrix are within the interval [0.2,0.4]; *constant signal propagation *is present when the ratio between the weakest and the strongest signal is smaller than 2; *asymmetric signal branching *and *asymmetric co-regulation *is present when there is more than a 20% difference between the signals coming to or arising from the system variables. Finally, *positive feedback *and *negative feedback *are described in the dependencies between the variables X3 and X2, and X5 and X2 respectively.

### 4.6 A note on the technical infrastructure and computational complexity of the experiments

To perform the GRN reverse-engineering experiments, we developed a module in Narrator [49] that provided an automatic interface to the Condor [50] distributed, high-performance computing system. This allowed computing tasks defined by Narrator to be automatically placed into the Condor scheduling queue. We used two pools of Condor computing clusters that were dedicated to our experiments: Pool 1 consisted of 23 Fujitsu Siemens E600 machines, each with a single Pentium 4 HT 3.06 GHz processor and 1 GB RAM; Pool 2 consisted of 10 Dell Optiplex GX 620 machines, each with a single Pentium 4 HT 3.4 GHz processor and 1 GB RAM. A typical evolutionary optimization would take about 35 minutes for the molecular models and 150 minutes for the network models. With the Condor pools we could repeat these runs a number of times, as this helped overcome problems due to the search process getting stuck in a local minimum. In total, it therefore took about 27 hours of compute time to perform a single reverse-engineering experiment. (For a 5-gene network with a detailed data set: 5 × 5 molecular model runs + 5 × 1 network model run = 25 × 35 min + 5 × 150 min = 875 min + 750 min = 1625 min.)

## Authors contributions

JM participated in conceiving and designing the experiments, performed some of the computational modelling, analyzed the results, and participated in drafting the manuscript. MS participated in conceiving and designing the experiments, performed some of the computational modelling, analyzed the results, and participated in drafting the manuscript. JM and MS contributed equally to this paper. WD participated in the design of the experiments and helped to draft the manuscript. All authors read and approved the final manuscript.
